# The Forgotten Skeletogenic Condensations: A Comparison of Early Skeletal Development Amongst Vertebrates

**DOI:** 10.3390/jdb7010004

**Published:** 2019-02-01

**Authors:** Jennifer L. Giffin, Danielle Gaitor, Tamara A. Franz-Odendaal

**Affiliations:** 1 Department of Biology, Mount Saint Vincent University, 166 Bedford Highway, Halifax, NS B3M 2J6, Canada; Jennifer.giffin@msvu.ca (J.L.G.); Dgaitor@dal.ca (D.G.); 2 Department of Medical Neuroscience, Dalhousie University, 5850 College Street, Halifax, NS B3H 4R2, Canada

**Keywords:** epithelial–mesenchymal interaction, condensation, bone, cartilage, skeleton, growth

## Abstract

The development of a skeletogenic condensation is perhaps the most critical yet considerably overlooked stage of skeletogenesis. Described in this comprehensive review are the mechanisms that facilitate skeletogenic condensation formation, growth, and maintenance to allow for overt differentiation into a skeletal element. This review discusses the current knowledge of gene regulation and characterization of skeletogenic condensations in the chicken, mouse, zebrafish, and other developmental models. We limited our scope to condensations that give rise to the bones and cartilages of the vertebrate skeleton, with a particular focus on craniofacial and limb bud regions. While many of the skeletogenic processes are similar among vertebrate lineages, differences are apparent in the site and timing of the initial epithelial–mesenchymal interactions as well as in whether the condensation has an osteogenic or chondrogenic fate, both within and among species. Further comparative studies are needed to clarify and broaden the existing knowledge of this intricate phenomenon.

## 1. Introduction

Condensations are the fundamental cellular units from which morphology is generated [1,2]. These densely-packed, round or ovoid-shaped aggregations of mesenchymal cells appear during the development of almost all tissues and organs, including the eye, ear, and nose sensory primordia, ectodermal appendages such as vibrissae, hair follicles and feathers, kidney and lung organ primordia, and musculoskeletal tissues, including muscle, ligaments, tendons, cartilage, and bone [3,4]. During skeletogenesis, condensations are the first identifiable sign that skeleton formation has started. Prior to condensation formation, an initial epithelial–mesenchymal interaction leads to the formation of a precondensation; this is followed by the development, growth, and maintenance of the condensation itself and, finally, the differentiation of osteo- and chondroblast cells within the condensation [4,5]. These steps are essentially the same for both cartilage and bone formation. Endochondrally ossifying bones will first form a cartilage template that will later ossify, while intramembranously ossifying bones form directly from the condensation. While skeletogenic condensations are often overlooked due to their transient appearance during embryonic development, they are just as important and identifiable as the future cartilaginous and bony structures they form [4]. Therefore, the primary aim of this review article is to provide an overview of the early stages of bone and cartilage development to facilitate a greater understanding of this critical developmental phase. 

Extensive research in skeletogenesis has been conducted in the chicken and mouse; however, a paucity of research exists for anamniotic vertebrates, such as fish and amphibians. Recent studies primarily reference the Hall–Miyake review papers [3,5,6] when characterizing early skeletogenesis. We implicitly assume that the early stages of bone development are equivalent amongst vertebrates. However, important differences among the vertebrate clades are apparent. For example, the molecular fingerprint of osteogenic condensations in land animals and ray-finned fish differs in the expression of Runt-related transcription factor 2 (*RUNX2*) and Sry-type HMG box 9 (*SOX9*) [7], two master regulatory genes of bone and cartilage differentiation, respectively [8]. *RUNX2* alone is expressed in mouse and chicken dermal bones [9,10,11], while both *runx2* and *sox9* are expressed in gar and zebrafish dermal bones [7,9,10]. Indeed, unique species-specific gene regulatory networks for cartilage and bone specification of neural crest cells are beginning to emerge (for a review, see References [12,13]). Thus, gene expression of skeletogenic tissues differs amongst species even in early developmental stages. Whether differences also exist in condensations prior to commitment to an osteogenic or chondrogenic fate has received less attention. It is also important to consider the cellular dynamics and the extracellular matrix (ECM) proteins necessary for condensation formation. Therefore, a second objective of this review paper is to compare the molecular signaling mechanisms and cellular attributes of growing skeletogenic condensations among different species. Comparative studies with different species allow for a deeper understanding of the key processes involved and provide insight into the evolutionary developmental history of vertebrates. 

## 2. Epithelial–Mesenchymal Induction: When, Where and How

The initial inductive epithelial–mesenchymal interaction may take place before, during or after migration of the presumptive skeletogenic cells from the neural crest or mesoderm to the future condensation site [4]. Rather than undergoing an epithelial-to-mesenchymal transition, epithelial–mesenchymal interactions involve a series of sequential and reciprocal communications between the epithelium and mesenchyme [14]. A series of seminal experiments led by Hall and colleagues in the mandible demonstrated the importance of timing in this interaction. For example, removal of the mandibular epithelium prior to Hamburger and Hamilton (HH) stage 24 in the chicken or embryonic day 10 in the mouse results in missing mandibular bones and/or cartilage, indicating a post-migratory interaction [3,15]. However, if isolated ectomesenchymal grafts at HH24 (embryonic day 10) or from earlier timepoints are recombined with epithelium at the same stage for several days, skeletogenesis ensues [3,15]. Meckel’s cartilage formation in the chicken mandible is induced by a cranial epithelial interaction with premigratory neural crest cells [16]; thus, it is not affected by epithelial removal at later stages [15]. Interestingly, isolated mandibular ectomesenchyme from the chicken and mouse was capable of responding to the epithelium of either species when recombined at HH22 in chicken embryos or embryonic day 9 in mouse embryos, although cartilage formation in the chicken ectomesenchyme only occurred when epithelium from embryonic day 10 mouse embryos was used [15]. Therefore, mandibular epithelial–mesenchymal interactions in the chicken and mouse embryo take place over a defined developmental period with all the necessary epithelial signaling factors, present at the outset or shortly thereafter. Remarkably, these signaling factors are conserved in both avian and mammalian species.

Precise timing of epithelial–mesenchymal interactions has also been studied in the chicken scleral ossicles, a ring of neural crest-derived bones situated at the corneal–scleral limbus that undergo intramembranous ossification in birds [17]. In this system, induction occurs after migration to the future condensation site, as scleral ectomesenchyme grafts did not form ossicles when isolated from the epithelium in chicken embryos at HH35 or younger but did after the completion of the induction period at HH36 [18]. A prolonged induction period is necessary, since epithelial–mesenchymal recombination experiments of HH35 or HH36 tissue are capable of forming bone, while HH31 mesenchyme incubated with HH35 epithelium is not [18]. Recent research has indicated that a maintenance period following the initial induction may begin as early as HH34, as early osteoblasts are detected by alkaline phosphatase staining at this stage [19]. During the extended induction period required for scleral ossicle development, the formation of specialized epithelial structures known as conjunctival papillae occurs; these papillae develop prior to but in a one-to-one ratio with the ossicles [20,21]. Recombinations of HH30-36 scleral ectomesenchyme with HH22 mandibular epithelium allowed for the formation of scleral ossicles with typical morphology [18], while recombinations of HH22 mandibular ectomesenchyme with HH31-35 conjunctival epithelium also formed normal mandibular bones, although the inductive ability of the conjunctival epithelium increased with age [18]. Altogether, these results indicate that post-migratory ectomesenchyme in the chicken is induced by the overlying conjunctival epithelium to form the scleral ossicles over an extended period of time and that the signaling molecules mediating this process are not specific to a particular epithelial site or tissue type, although greater efficiency is attained as the inductive period proceeds. 

The location of epithelial–mesenchymal interactions during mandibular arch development has been studied in other species, including urodele amphibians and teleost fish. The pharyngeal endoderm has been shown to induce migrating neural crest cells in the Alpine newt, as co-cultures of these cells result in cartilage formation [22,23]. Similarly, interaction between the pharyngeal arch and migratory neural crest cells exists in zebrafish, as mutant zebrafish larvae that lack the pharyngeal pouches have greatly reduced pharyngeal cartilage [24], while grafting of wildtype cells into endoderm-deficient mutants restores cartilage formation [25]. While scleral ossicles are present in both teleosts and reptiles [17], the chicken remains the only species for which the location of epithelial–mesenchymal interactions is currently known (reviewed in [26]). Determining the location as well as the inductive capabilities and the developmental timeframe of epithelial–mesenchymal interactions in the craniofacial region are therefore areas of future research for emerging model organisms. Clearly, differences in the timing of neural crest cell migration amongst species are present; however, the signaling molecules involved appear to be more conserved. 

Several inductive epithelial signaling molecules required for initiation of mesenchymal condensations have been identified, including bone morphogenetic protein (*BMP*) 2 and muscle segment homeobox (*MSX*) 1, a target gene of *BMP* [27], transforming growth factor β (*TGFβ*), and tenascin [2,5]. These signaling factors may be introduced to the mesenchyme in three main ways: direct cell–cell interaction, through the basement membrane or via diffusion [4]. Interestingly, a very close proximity of osteogenic condensations to the overlying epithelium has been reported in the mandible of chicken embryos [3] and this has become a defining characteristic of osteogenic condensations [28,29], suggesting that closer cell–cell or ECM interactions may be more common among this condensation type. Indeed, a matrix-mediated mechanism of epithelial–mesenchymal interaction has been described for osteogenic as well as chondrogenic mandibular condensations in chicken and mouse embryos [30]. However, diffusion-mediated signaling is a functioning mechanism in osteogenic condensations, as seen in the chicken scleral ossicles [31]. 

Supporting evidence for the involvement of the above signaling molecules has been obtained over the past several decades, for both osteogenic and chondrogenic condensations. *BMP* and fibroblast growth factor (*FGF*) are potential inductive factors in the chicken scleral ossicle system [31,32,33,34]. During the onset of scleral ossicle induction at HH35, *BMP2* was localized to the conjunctival epithelium and upon inhibition by Noggin bead implantation, a gap in the scleral ossicle ring where the bead had been implanted was found [33]. In chondrogenic condensations, however, *BMP* appears to function primarily after condensation formation has taken place. This was demonstrated in micromass cultures of chicken limb buds, in which cartilage nodules increased in size but not number upon application of BMP2; however, the number of cartilage nodules did increase with TGFβ application [35]. The inductive role of *TGFβ* in chicken limb buds was further demonstrated when beads placed in the interdigital space bearing TGFβ-1 or -2 initiated formation of ectopic cartilages or extra digits, whereas limb buds with beads bearing BMP4 did not [36]. The effects of *TGFβ* may be mediated by several adhesion molecules, including N-cadherin and neural cell adhesion molecules (NCAM), as well as ECM molecules, such as fibronectin and tenascin [37]. Tenascin, a matrix glycoprotein, and syndecan, its cell surface receptor, have been suggested to play a key role in facilitating epithelial–mesenchymal interactions based on their presence and timing in both osteogenic and chondrogenic condensations [3,37]. Furthermore, the addition of tenascin to chick limb bud micromass cultures results in a higher number of cartilage nodules, indicating its involvement in promoting condensation formation [38]. The hedgehog (*HH*) family of signaling molecules may also have a similar function in both chondrogenic and osteogenic condensations. Missing or enlarged scleral ossicles were observed in the chicken embryo after implanting beads soaked with cyclopamine, an inhibitor of the *HH* co-receptor Smoothened, into the conjunctival epithelium at HH35 or 36 [32]. Additionally, in mutant mice with sonic hedgehog (*Shh*) inhibition in the epithelium of the first pharyngeal arch, a complete loss of chondrogenic condensations in the mandibular arch was found; however, osteogenic condensations still formed [39], suggesting that different matrix-mediated signaling mechanisms may be present for cartilage and bone development in the mouse lower jaw. In summary, while both osteogenic and chondrogenic condensations are induced to form by the same signaling mechanisms, some signaling molecules such as *BMP* and *TGFβ* may not have the same function, while others such as tenascin or HH family members may be similar.

Further evidence to support the role of *BMP2* and *SHH* in mediating epithelial–mesenchymal interactions has been demonstrated in the zebrafish. Ectopic expression of *bmp2* or *shh* in the regenerating fin blastema induced excess dermal bone deposition in the inter-ray region, which is normally devoid of bone [40]. Furthermore, inhibition of *bmp* with Chordin during fin regeneration led to a decrease in blastema proliferation and fin outgrowth in addition to downregulation of *msx* genes [41]. However, since regeneration occurs only in the dermal fin rays and not the endoskeleton cartilage [41], it is unknown whether these signaling molecules have the same function in both chondrogenic and osteogenic condensations. Therefore, the role of BMP and HH family members appears conserved in osteogenic condensations in avian, mammalian, and teleost species. 

## 3. Initiating and Maintaining Skeletogenic Condensations

**Figure 1:**
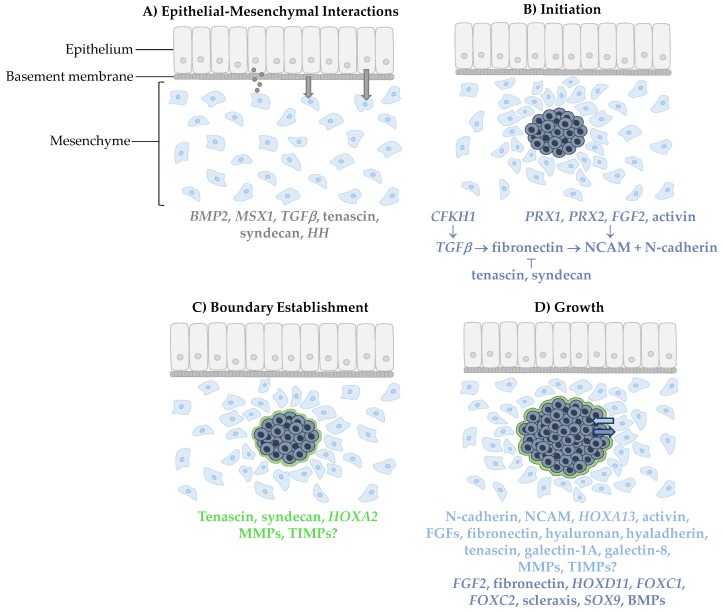
Condensation formation. (**A**) Mesenchymal cells are first induced by epithelial signaling molecules through diffusion, close interaction with the basement membrane or direct cell–cell interaction. (**B**) Condensation is then initiated by a signaling pathway involving neural cell adhesion molecules (NCAM) and N-cadherin. (**C**) A boundary surrounding the condensed cells is then established which permits (**D**) growth of the condensation from incoming cells that adhere to the condensation and cells undergoing proliferation within the condensation. Signaling molecules involved in each step are color-coded for the location in which they appear. Grey, dark blue, green, and light blue correspond to epithelial cells, condensed cells, the condensation boundary, and mesenchymal cells, respectively.

Upon induction of the mesenchyme to form a condensation, the mesenchyme signals back to the epithelium in a positive feedback loop as well as within the mesenchyme itself for precise regulation of the condensation process [4]. *FGF2*, *TGFβ*, fibronectin, NCAM, chicken forkhead helix 1 (*CFKH1*), and paired-related homeobox (*PRX*) 1 and 2 have been identified as major genes or gene products associated with initiating skeletogenesis [2]. Additionally, activin, a member of the *TGFβ* family, has also been shown to induce condensation formation in the chicken limb bud [42]. Ultimately, these signaling molecules act by upregulating NCAM, which functions in the stabilization and maintenance of condensations [2]. N-cadherin, a cell–cell adhesion molecule involved in adhesion [43] and communication via gap junctions [44], is co-expressed with NCAM. Tenascin and syndecan are also involved at this stage by binding to fibronectin, which inactivates NCAM, thus inhibiting condensation formation [2]. A functional summary of these molecules is presented in Figure 1 and Table 1.

Condensations at this stage can be identified by staining for particular proteins, such as peanut agglutinin (PNA), a galactose-specific lectin that selectively binds the cell membranes of early chondrogenic condensations in mouse and chicken limb buds [45,46]. PNA has also been used to stain chondrogenic and osteogenic condensations in the first arch of mice and chicken, respectively [3,47]. However, greater specificity for osteogenic condensations can be obtained with alkaline phosphatase and *RUNX2* expression localization [29]. PNA stains condensations before the deposition of cartilage-specific ECM materials [45,48] around the time they first become visible in vitro [48]. However, earlier markers of chondrogenic condensations have been identified, such as galectin-1A and galectin-8 [49]. Galectin-1A and galectin-8 also play a role in regulating condensation size [49], indicating a very close temporal relationship among subsequent stages of condensation growth. Reliance on well-known stains such as alizarin red for the identification of ossification centres is not accurate for assessing bone homologies amongst organisms (e.g., [50]) due to the ability of single condensations to develop into more than one ossification center [51,52,53]. Additional markers of condensation initiation stages are needed, especially in future bone-forming regions.

## 4. Setting Condensation Boundaries

Establishing condensation boundaries is an important early step of condensation formation, with changes in location occurring throughout subsequent growth and development. Thus, it is not surprising that some of the molecules involved in condensation initiation and growth, including tenascin, syndecan, and homeobox A2 (*HOXA2*), also play a role in establishing the condensation boundaries [2]. Tenascin and syndecan surround chondrogenic and osteogenic condensations in a complex spatiotemporal pattern [37,38,54]. In addition to their localization to the condensation boundary, a role for tenascin and syndecan can be inferred based on their relationship with other ECM molecules. As the known substrate for several matrix metalloproteinases (MMPs), enzymes involved in ECM remodeling [55], tenascin may act as a negative regulator for condensation growth. Thus, MMPs and their counteracting tissue inhibitors (TIMPs) could be useful candidates to study condensation boundary formation and degradation. A similar pattern of *Hoxa2* surrounding the condensations has been demonstrated in the second branchial arch in mice [56]. HOXA2 has been shown to regulate morphogenesis of skeletal elements by establishing chondrogenic and osteogenic domains. In *Hoxa2* null mutant mice, *Sox9* expression in the second branchial arch was shifted and osteogenic condensations, which do not typically form, were present [56]. A role for *Hoxa2* in dermal bone inhibition was mediated by expression of core-binding factor 1 (*Cbfa1*) [56], also known as *Runx2* [29], as observed in osteogenic condensations of the murine palatal process [57]. Therefore, establishing condensation boundaries requires a delicate balance of containing cells while still allowing cells to enter the condensation as mediated by tenascin and syndecan, with a potential role for MMPs (Figure 1, Table 1). Additionally, osteogenic and chondrogenic domains must be properly maintained by *HOXA2* to allow for normal morphology.

## 5. Condensation Growth

The central dogma of condensation growth is that before bone or cartilage formation can occur, the skeletogenic condensation must reach a critical size [2]. Condensation growth is achieved by cellular activities that may affect mesenchymal cells both inside and outside the condensation: Cell proliferation, cell death, cell cycle, cell movement, and cell aggregation towards a central point [2,5,6,53]. Condensation size may also be affected by extrinsic mechanisms, such as mechanical and endocrine factors [2], or by a local activation lateral inhibition (LALI) self-organizing system [5,6] (for a review, see Reference [58]). Concurrently with condensation growth, patterning of the condensation in shape, number, arrangement, and location takes place and establishes the future skeletal morphology [7,59]. 

### 5.1. Condensation Adhesion and Cell Migration

The migration of cells towards a center or lack of cell movement away from a center is the predominant mechanism of skeletogenic condensation growth for the majority of species [53]. N-cadherin, NCAM, and *HOXA13* are recognized for their roles in promoting adhesion [2], which recruits the surrounding mesenchymal cells into the condensation. Without these signaling molecules present, a reduction in size but not a complete absence of condensation formation is observed. For example, condensation size in a chicken limb bud micromass decreased when exposed to an NCAM antibody but increased with electroporation of *NCAM*-containing plasmids [60], or upon exposure to activin [42]. Similarly, condensation size and uniformity decreased in chicken limb bud cell cultures transfected with deletion mutants of N-cadherin, while cultures over-expressing N-cadherin had normal condensation formation but were unable to proceed toward differentiation [43]. Unexpectedly, in whole organ cultures of transgenic mouse limb buds deficient in N-cadherin, condensation and chondrogenesis occurred normally; however, this could be explained by the greater ability of whole organ systems to compensate for disruption in protein functions with other cell–cell adhesion molecules, such as cadherin-11 [61]. In chicken limb buds infected with a *HOXA13* recombinant virus, condensation size was reduced and histomorphology of the resulting bone was altered by a homeotic transformation from long bone to short bone [62]. Limb bud patterning was also affected in *Hoxa13* mutant mice, which demonstrated fewer, smaller, and less robust condensations, resulting in some missing autopod elements [63]. FGFs may also have a role in promoting cell adhesion, as N-cadherin expression was disrupted by the pharmacological application of FGF receptor (FGFR) inhibitors in the chicken limb bud [64]. Therefore, adhesion molecules play an important role in condensation growth and patterning, and their function can be compensated for by several related molecules. 

In addition to their involvement in earlier steps of the condensation process, a variety of extracellular matrix molecules, including fibronectin, hyaluronan, hyaladherin, and tenascin are important regulators of condensation size [2,4,5]. When embryonic chicken limb bud cultures were treated with fibronectin antibody or hyaluronan hexasaccharide, a loose organization or delayed appearance of condensations with reduced number and sizes occurred [48,65]. Recently, cell adhesion mechanisms in conjunction with a LALI network have been identified [58]. In the embryonic chicken limb bud, galectin-1A and galectin-8 have been shown to interact such that galectin-1A acts as a local activator of condensation through its adhesive properties and galectin-8 acts as a long-range inhibitor of galectin-1A action [48]. This system is considered a Turing-type reaction–diffusion mechanism [66]. A mechanochemical LALI mechanism has also been demonstrated in the pharyngeal arches of the embryonic chicken [67], involving mechanical interaction of the cells with the surrounding ECM, as well as signaling molecules [58]. MMPs and TIMPs are involved in modulating the diffusion properties of morphogens [55,68]; therefore, they may also have a role in the fine-tuning of LALI models. These self-organizing mechanisms of development have been relatively unstudied until recent years but hold great potential for providing new insights into the condensation process. The involvement of the ECM in condensation adhesion is therefore an expanding area of research with contributions from both cellular and physicomechanical factors. 

### 5.2. Condensation Proliferation

While condensation size may be influenced by cell proliferation or cell death [2,5,6], this does not appear to be the predominant mode of condensation growth in skeletogenic systems, such as the avian limb bud [69,70] or scleral ossicles [32,71]. However, the rate of cell proliferation/cell death (cell cycle) has been shown to influence the developing avian mandible. When quail ectomesenchyme was transplanted into duck hosts, a smaller beak size, earlier expression of osteogenic genes, and mineralization was observed, consistent with the quail donor’s faster cell cycle [72]. Therefore, cell proliferation and cell death could have considerable importance in condensation growth. 

A role for *FGF2*, fibronectin, *HOXD11*, mesenchymal forkhead-1/forkhead box C2 (*MFH-1/FOXC2*), scleraxis, and *SOX9* in cell proliferation has been described previously [2]. Recent supporting evidence for the proliferative role of several of these signaling molecules has been provided in transgenic murine models. In mice with inactivated *Fgfr2* in the apical ectodermal ridge or *Hoxa11/Hoxd11* double mutant mice, a decrease in proliferation as well as an increase in apoptosis resulted in severely shortened limb buds without any subsequent cartilage formation [73,74]. A similar phenotype was observed in mutant mice when *Sox9* was inactivated in undifferentiated mesenchymal cells as a result of increased apoptosis [75]. In addition to *Foxc2*, a role for *Foxc1* in the proliferation of calvarial mesenchymal cells was shown in *Foxc1*-null mutant mice [76]. *Foxc1* has also been shown to mediate the *Bmp*-induced upregulation of *Msx2* in the murine frontal bone [77], suggesting a role for *Bmp* in condensation proliferation in addition to its inductive role on the mesenchyme.

BMPs are recognized for their role in condensation growth [2] and are key players in the regulation of cell proliferation and survival. In a comparative study of Darwin’s finches, higher and earlier expression of *BMP4* was found in species with deeper, broader beaks, and this phenotype was induced in other species with smaller beaks upon infection with *BMP4* retrovirus [78]. Additionally, in chickens infected with viruses expressing *BMP2* or *BMP4*, condensations in the lower jaw were larger as a result of increased cell proliferation [79]. When *BMP2* and *BMP4* were inhibited by infection with Noggin in the chicken embryo, condensation formation in the frontal bone did not progress to differentiation as detected by the absence of *RUNX2* expression [80]. Additionally, when a Noggin-soaked bead was placed into the maxillary bone of chicken embryos, there was a significant decrease in cell proliferation and an increase in apoptosis in the developing condensations [81]. Similarly, in the limb buds of mutant mice with loss of *Bmp*-*Smad* signaling, an increased rate of apoptosis was observed in condensations, and differentiation did not occur [82]. Notably, expression of cell surface adhesion molecules *cadherin 2* and *NCAM1* and *2* was still present in micromass cultures at normal or higher than normal levels [82], suggesting that while *BMP2* promotes the expression of N-cadherin during chondrogenesis [83], other signaling molecules take part, since condensation as a multistep process was not entirely suppressed. Furthermore, in *Noggin* mutant mice with *Bmp* gain-of-function signaling, the proliferation rate of mesenchymal cells in the Meckel’s cartilage condensations was greatly enhanced [84]. Interestingly, Meckel’s cartilage in the above study did not degenerate as normally occurs in mammals but rather underwent endochondral ossification and formed part of the developing mandible [84]. Similarly, cartilage formation in the chicken was induced when a Noggin-soaked bead was implanted into the maxillary bone, which normally undergoes intramembranous ossification, at HH15 but not HH20 [81], suggesting that *BMP* acts in a dose- and time-dependent manner on the survival and fate of condensations. Therefore, *BMP* appears to be intricately involved in skeletogenic condensation growth, by enhancing proliferation and/or suppressing death of the mesenchymal cells. Furthermore, the timing and level of *BMP* expression is capable of directly influencing cell fate. 

## 6. Conclusions

Prior to differentiation into mature bone or cartilage, the condensations of skeletal tissues are responsible for establishing the tissue histomorphology, location, size, and shape of the future skeletogenic element. The initial inductive signaling from the epithelium occurs in a precise spatiotemporal manner, with some well-conserved molecules among species, tissues, and epithelial sites, such as tenascin and the *HH* gene family. A well-known signaling pathway leading to the expression of NCAM and N-cadherin allows for condensation formation, which can be visualized with several gene or protein markers. Boundaries are then formed to maintain the condensation as a separate entity from the surrounding mesenchyme, giving it a unique identity. However, ongoing remodeling of the boundaries in the ECM takes place to allow for the further recruitment of cells into the condensation such that it can reach a critical size. Condensation growth may also occur by cell proliferation and enhancing cell survival rates within the condensation. Numerous signaling molecules are involved in increasing condensation size; however, the underlying genetic pathways remain to be elucidated. Patterning of the condensation occurs while it grows by various emerging mechanisms such as, for example, LALI networks. Thus, the formation and regulation of skeletogenic condensations is a complex and dynamic process with crucial importance for all subsequent phases of development. 

Although the fundamental stages in condensation formation are known, much remains to be discovered about the molecular regulation of the different stages and the conservation of skeletogenic condensation formation and growth across vertebrates. A role for *HH* family members in mediating epithelial–mesenchymal interactions and for *FGF* and *BMP* in promoting cell adhesion and cell proliferation, respectively, within condensations has recently emerged. However, very few condensation boundary markers are currently known; consequently, there is an incomplete understanding of how cells migrate into condensations while maintaining established boundaries. Several molecules are known to be involved at multiple stages, but definable molecular markers are largely lacking. A surprisingly limited number of skeletogenic condensations have been studied in the chicken and mouse models, and even fewer in the fish model. Even after several decades of research in this field, it is evident that more studies are needed to bridge the many gaps in knowledge of the early stages of skeletal element development. Understanding condensation formation can ultimately shed light on many aspects of the later stages of skeletogenesis and can unveil some of the developmental and mechanistic events that lead to skeletal anomalies and define evolutionary relationships among vertebrate species.

## Figures and Tables

**Table 1:** Active signaling molecules and their function at different stages of condensation formation.

Stage	Gene/Gene Product	Function
Epithelial–Mesenchymal Interactions	*BMP2*	Inductive epithelial signaling molecules
	*MSX1*	
	*TGFβ*	
	Tenascin	
	Syndecan	
	*HH*	
Initiation	*CFKH1*	Regulates TGFβ
	*TGFβ*	Regulates fibronectin
	Fibronectin	Regulates NCAM
	*PRX1*, *PRX2*	
	*FGF2*	
	Activin	
	Tenascin	Adhesion; inhibits fibronectin
	Syndecan	
	NCAM	Adhesion; stabilization and maintenance of condensation
	N-cadherin	Adhesion
Boundary Establishment	Tenascin	Boundary formation
	Syndecan	
	MMPs, TIMPs	Potential role in boundary degradation
	*HOXA2*	Establishment of osteogenic and chondrogenic domains
Growth	N-cadherin	Cell adhesion
	NCAM	
	Activin	
	FGFs	
	Fibronectin	
	Hyaluronan, hyaladherin	
	Tenascin	
	*HOXA13*	Cell adhesion and condensation patterning
	Galectin-1A, galectin-8	
	*FGF2*	Cell proliferation and survival
	*HOXD11*	
	*FOXC1*	
	*FOXC2*	
	Scleraxis	
	*SOX9*	
	BMPs	
	MMPs, TIMPs	Potential role in boundary degradation to allow growth
